# Anatomical insights into *the peri-trigeminal zone* via transorbital, transclival, and retrosigmoid routes: a comparative cadaveric study with surgical implications

**DOI:** 10.1007/s00701-026-06789-4

**Published:** 2026-02-28

**Authors:** Bruno Vernile, Marianna Di Costanzo, Alejandra Mosteiro, Marta Codes, Gloria Cabrera, Andrès Apolinar, Francesco Sala, Barbara Masotto, Joaquim Enseñat, Alberto Di Somma, Alberto Prats Galino

**Affiliations:** 1 University of Verona, Verona, Italy; 2 University of Naples Federico II, Naples, Italy; 3 Departament de Cirurgia i Especialitats Medicoquirúrgiques, Facultat de Medicina i Ciències de La Salut, Universitat de Barcelona (UB), Barcelona, Spain; 4 Institut Clínic de Neurociències (ICN), Department of Neurological Surgery, Hospital Clínic de Barcelona, Barcelona, Spain; 5 Institut d’Investigacions Biomèdiques August Pi i Sunyer (IDIBAPS), Universitat de Barcelona (UB), Barcelona, Spain; 6 Neurosurgery Department, Posterior Cranial Fossa Unit, University Hospital of Verona, Borgo Trento, Verona, Italy

**Keywords:** Peritrigeminal zone, Brainstem, Endoscopic, Transorbital, Skull base

## Abstract

**Background:**

Surgical access to brainstem (BS) lesions requires small neurotomies in between a dense and complex neural fiber network. Access is gained on the BS surface closest to the lesion to minimize the intraparenchymal trajectory and reduce the risk of neurological injury. The concept of safe-entry zones guides the selection of the most favorable entry point to reduce these risks. Several endoscopic approaches have been validated as safe and effective for accessing the *peritrigeminal zone* (PTZ); however, each one is limited by anatomical constraints due to adjacent osteo-vascular structures, which restrict the surgical corridor. To evaluate the anatomical advantages and limitations of accessing the PTZ via the *endonasal transclival approach* (ETTA), *retrosigmoid approach* (RS), and *endoscopic transorbital approach* (ETOA).

**Methods:**

The ETTA, RS, and ETOA approaches were performed on five human cadaveric specimens (25 approaches). Before dissection, all specimens underwent high-field magnetic resonance imaging, including *diffusion tensor imaging* (DTI) sequences for tractography reconstruction. An anatomical assessment was then conducted to verify accessibility to the PTZ. The potential surgical trajectory, approach length, and surgical view’s angle were measured and compared across the three approaches.

**Results:**

All approaches allowed access to the PTZ; however, each one exhibited structural limitations affecting surgical maneuverability. Comparative anatomical and radiological analysis highlighted procedural insights to guide the selection of the most appropriate surgical corridor based on lesion morphology. The RS approach, the shortest one, and the ETTA provided a near-tangential visualization of the PTZ, whereas the ETOA offered a more perpendicular surgical view.

**Conclusions:**

A thorough understanding of the anatomical and technical nuances of the three approaches to the PTZ described in this study can support the selection of the most appropriate surgical route for pontine lesions. Comparative data suggest that the orientation of the lesion’s major axis within the pons is a key criterion in determining the optimal surgical approach.

## Introduction

Surgical access to the brainstem (BS) represents one of the greatest challenges in neurosurgery due to its dense network of critical vascular and neural structures. Despite advances in surgical anatomy knowledge and technological tools, access to the BS remains complex and carries significant risks.

To minimize postoperative morbidity, the so-called BS *safe entry zones* have been designed to enable circumferential approaches to various sites on the BS surface without eloquent neural structures [6, 7, 38]. In the era of intraoperative neurophysiological monitoring, the ability to map neural tracts has further reduced these risks [31, 36]. The advent of endoscopic and endoscope-assisted microsurgery has significantly expanded the surgical armamentarium in this area, allowing access to the anterior and lateral regions of the BS. In addition to the well-known posterior safe entry zones, relatively safe anterolateral entry areas have also been identified [7, 38, 44].

Among the more anterolateral safe entry zones of the brainstem is the *peritrigeminal zone* (PTZ)*,* defined as the region bounded posteriorly by the *root-entry zone* of the trigeminal nerve and the *root-exit zone* of the facial nerve, and the pons’ prominence of pyramidal tract anteriorly. This zone is medially bordered by the corticospinal tract, inferiorly by the intrapontine fibers of the facial nerve, laterally by the intrapontine fibers of the trigeminal nerve, and deeply by the trigeminal motor nucleus and spinal tract [7, 38]. Injury to these adjacent structures may result in contralateral hemiparesis, trigeminal sensorimotor dysfunction (e.g., altered facial sensation, neuralgia, or masticatory disturbances), and ipsilateral facial paresis.

Access to the anterolateral BS has been achieved through approaches such as the retrosigmoid, presigmoid retro-labyrinthine (with or without tentorial incision), trans-labyrinthine, and, for more ventral targets, skull base approaches like the orbitozygomatic or pterional routes via a pretemporal corridor with the removal of the petrous apex to reach the posterior cranial fossa [2, 6, 19, 26, 31, 39, 39, 40, 46].

However, the literature now includes anatomical and surgical studies demonstrating the feasibility of accessing the anteroventral brainstem via extended endoscopic transnasal-transclival approaches, with or without anterior petrosectomy [10, 16, 20, 27, 33, 42]. These techniques offer a closer view of the pons, reduce parenchymal retraction, decrease residual tumor volume, and in some cases, reduce the area required for myelotomy.

In the last decade, the transorbital endoscopic approach has emerged as a safe and valid option for accessing the posterior fossa via a “pretemporal route” associated with extradural anterior petrosectomy [14, 21, 28].

Therefore, access to the PTZ can be achieved through anterior, lateral, or posterior surgical corridors. Improving surgical efficacy and reducing the risk of recurrence for intrapontine lesions involves selecting an approach based on the lesion’s morpho-volumetric characteristics. The ideal strategy follows the "one-point method" concept, in which the entry point is as close as possible to the lesion's margin and aligned with its major development axis [35].

The anatomical study aims to evaluate the limitations of endoscopic transsphenoidal-transclival, retrosigmoid, and transorbital approaches for reaching the peritrigeminal zone, and to demonstrate the surgical dissection trajectories each approach offers.

## Materials and methods

This study was conducted in accordance with ethical guidelines for anatomical research. The cadaveric specimens used for dissection were obtained through legally authorized donation programs, with prior informed consent provided by the donors or their next of kin. All procedures were performed in compliance with institutional and national ethical regulations, including the Declaration of Helsinki (2013), ensuring respect for the dignity and integrity of the donors.

### Anatomical dissections and qualitative analysis

Anatomic dissections were performed at the Laboratory of Surgical Neuroanatomy (University of Barcelona, Spain). Five human head specimens (25 approaches: 10 orbits, 10 retrosigmoid approaches, 5 nasal cavities) were cleaned of blood clots, fixed with Cambridge solution, and injected with red latex into the common carotid arteries. Before the dissection, all specimens underwent a high field magnetic resonance imaging study (3 T) with diffusion-tensor-imaging (DTI) sequences with subsequent tractography (Brainlab Elements Fibertracking module—Brainlab AG, Munich, Germany).

Endoscopic transsphenoidal transclival, endoscopic transorbital approaches were performed using a 4 K rigid endoscope of 4 mm diameter and 15,5 cm length, with 0° lenses (Stryker Endoscopy, San Jose, California, USA). After performing a craniectomy in the retrosigmoid region, a 0° lens endoscope was used to assess the peritrigeminal area. A 4 K endoscopic camera system (Stryker, Kalamazoo, MI, USA) was utilized in conjunction with a fiber-optic LED light source to perform the endoscopic procedures.

After dissection, the direction followed by each of the three approaches was captured with a navigation probe by the BrainLab navigation system (Brainlab AG, Munich, Germany), with the surgical target set at the medial margin of the root-entry zone of the trigeminal nerve. An evaluation of the distance from the skin to the target, to simulate the surgical length, was carried out on the pre-dissection MRI studies.

Finally, to provide a comparative anatomical representation of the different approaches to the brainstem surface at PTZ, a formalin-fixed and subsequently frozen human brainstem was isolated and stored at –15 °C for two weeks, then prepared according to Klingler’s fiber dissection technique [45].

### Radiological assessment of the optimal surgical trajectory and entry zone

To evaluate the surgical viewing angle, preoperative planning was performed using the Brainlab navigation system (Brainlab AG, Munich, Germany). A virtual trajectory was defined from the midpoint of the planned craniotomy at the skin level to the midpoint of the target area on the ventral surface of the pons. The target area was anatomically delineated between the root entry zone (REZ) of the trigeminal nerve and the root exit zone (REZ) of the facial nerve posteriorly, and anteriorly by the corticospinal tract as visualized on preoperative tractography or anatomical MRI landmarks.

A second reference trajectory was aligned tangential to the surface of the pons. The angle between the working trajectory and the tangential vector to the pontine surface, the virtual “*surgical view’s angle*”, was calculated using Brainlab's built-in measurement tools.

### Statistical analysis

The surgical viewing angle and the length of each approach were computed and data were tested for normality prior to further analysis. A one-way analysis of variance (ANOVA) was performed to assess for statistically significant differences in mean surgical corridor lengths between the three approaches. Post hoc pairwise comparisons were carried out using Bonferroni correction. A *p*-value < 0.05 was considered statistically significant. All analyses were performed using Python (v3.10) with the statsmodels and scipy libraries.

### Endoscopic retrosigmoid approach (RS)fig

The simulated surgical procedures were conducted with the patient in the lateral decubitus position. A C-shaped retroauricular skin incision was performed, followed by subperiosteal dissection of the suboccipital muscles to expose key anatomical landmarks including the asterion, the inferolateral surface of the occipital bone, and the mastoid tip. A retrosigmoid craniectomy was then carried out using a high-speed drill, exposing the transverse and sigmoid sinuses. The dura mater was incised in a C-shaped manner, based on the contour of the transverse and sigmoid sinuses, and reflected posteriorly to allow dural retraction and access to the cerebellopontine cistern. The endoscope was then progressively advanced between the cerebellar surface and the petrosal bone, and the CPA was completely explored.

### Endoscopic trans-sphenoidal-trans-clival approach (ETTA)

An extended endoscopic endonasal approach to the clivus was performed as previously described [8, 12, 25]. The heads were positioned in approximately 10 degrees of cervical extension to replicate operative conditions and with a 0-degree rigid endoscope. To gain more working space, a middle meatal antrostomy was performed. A standard sphenoidotomy was performed to access the sphenoid sinus, followed by meticulous drilling of the sphenoid floor, extending laterally to the Vidian canals, which defined the lateral boundaries of the exposure. The paraclival segments of the internal carotid arteries (ICAs) were fully skeletonized bilaterally to establish the surgical corridor. Superiorly, the approach was extended to expose the sellar region, the parasellar carotid genu, and the medial opticocarotid recess. Inferior dissection proceeded to the level of the abducens nerve porus, which delineated the inferior limit of the surgical field. After lateral mobilization of the paraclinoid ICA segment, drilling advanced to the entrance of Dorello’s canal, considered the lateral limit of the dissection. A Y-shaped dural incision was fashioned, with the vertical limb oriented parallel to the course of the basilar artery, and the horizontal limbs following the contour of the sellar floor. This configuration allowed for anterior reflection of the dural folds to expose the ventrolateral aspect of the pons.

### Endoscopic transorbital approach (ETOA)

A superior eyelid endoscopic transorbital approach with extension to the petrous apex was performed as previously described [11, 13, 15]. The cadaveric heads were positioned supine, with a slight rotation of 10–15° toward the contralateral side. The lateral orbital rim was also removed to increase the working space and facilitate access to and drilling of the middle cranial fossa floor. After reaching the petrous apex, a medial mobilization of the Gasserian ganglion–mandibular nerve complex was performed to improve exposure of Kawase’s triangle. Using a high-speed diamond burr, the petrous apex was drilled down to the anterosuperior wall of the internal auditory canal. Following the opening of the dura mater of the posterior cranial fossa, the PTZ was accessed.

## Results

All three approaches allowed for an extensive exposure and visualization of the PTZ.

Via the retrosigmoid approach, the PTZ area is identified in an inferomedial position relative to the surgical view, which proceeds in a postero-anterior and supero-inferior direction. The transsphenoidal-transclival route, by contrast, provides an anterolateral perspective of the PTZ, with an antero-posterior projection. The transorbital approach enables an anterior and more direct visualization of the region, with an antero-lateral projection.

**Fig. 1:**
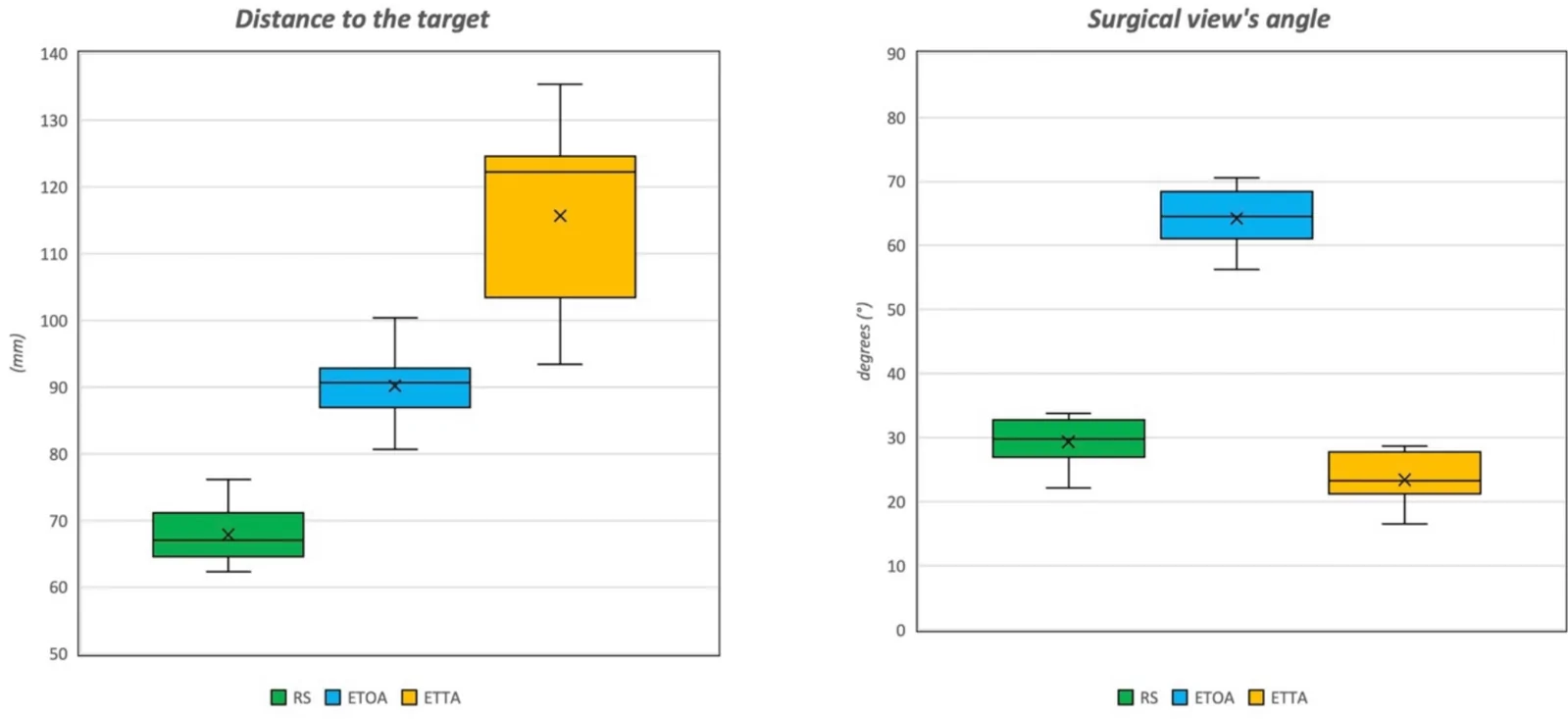
Results. On the left, box-plot showing the distribution of surgical corridor lengths for the RS, ETOA, and ETTA approache; on the right, the box plot show the distribution of the surgical view’s angles for the three appraches. Boxes represent the interquartile range (IQR), with the horizontal line indicating the median. Whiskers extend to 1.5 × IQR, and dots indicate outliers

As discussed in the following paragraphs, the RS, the shortest route, and ETTA approaches provide a dissection trajectory tangential to the PTZ, whereas the ETOA offers an almost perpendicular view of the PTZ. A comparative visualization of the dissection trajectories afforded by these three surgical corridors has enabled an enhanced three-dimensional anatomical understanding of the PTZ (Fig. 1).

Table 1 provides a synthesis of the potential advantages and limitations associated with the retrosigmoid, endoscopic transsphenoidal–transclival, and transorbital routes based on our anatomical findings.

**Table 1:** Advantages and limitations associated with the approaches

Approach	Advantages	Limitations
Retrosigmoid approach (RS)	- Shortest surgical corridor (~ 68 mm); - Relatively physiological trajectory through the cerebellopontine angle (CPA); - Requires limited bone removal; - Provides reliable exposure of the trigeminal and facial nerve root entry/exit zones	- Tangential trajectory to the pontine surface, reducing surgical maneuverability; - May necessitate cerebellar retraction or suprameatal tubercle drilling; - Limited access to ventral or medial pontine lesions; - Risk of injury to CPA structures, (facial–vestibulocochlear bundle)
Endoscopic trans-sphenoidal trans-clival approach (ETTA)	- Provides anterior/anterolateral access to the pons; - Minimize cerebral retraction; - Enables closer working distance to ventral lesions into the pons	- Longest surgical corridor (~ 116 mm); - Tangential approach to the brainstem impairs direct visualization of the peritrigeminal zone; - Requires extensive clival drilling and bilateral carotid skeletonization - Risk of injury to the basilar artery, perforating branches, and abducens nerve - Surgical field constrained by the ICA laterally and the pontine parenchyma medially
Endoscopic trans-orbital (ETOA)	- Intermediate surgical corridor (~ 90 mm) - Provides a near-perpendicular trajectory to the pontine surface; - Direct visualization of both trigeminal and facial nerves; - Reduces temporal lobe retraction compared with traditional transcranial routes	- Requires anterior petrosectomy with drilling of Kawase’s triangle; - Potential morbidity related to the middle meningeal artery and greater superficial petrosal nerve - Risk of cavernous sinus injury during lateral wall manipulation - Technically demanding, requiring advanced endoscopic expertise

### Qualitative anatomical assessment

The retrosigmoid corridor provides a direct line of sight to the PTZ following meticulous arachnoid dissection, which decompresses the CPA up to the exposure of the facial-vestibulocochlear bundle. Dissection proceeds toward Dandy’s vein, located superomedially, and subsequently toward the trigeminal nerve root entry zone (REZ).

Although the inclination of the petrous surface of the temporal bone directs the surgical view toward the trigeminal REZ, a full exposure of the PTZ, aligned with the endoscopic axis, may require retraction of the cerebellar parenchyma, and occasionally of the superior cerebellar peduncle or the cerebellar flocculus. A prominent suprameatal tubercle may hinder visibility, in which case drilling of the tubercle can facilitate a wider operative window.

**Fig. 2:**
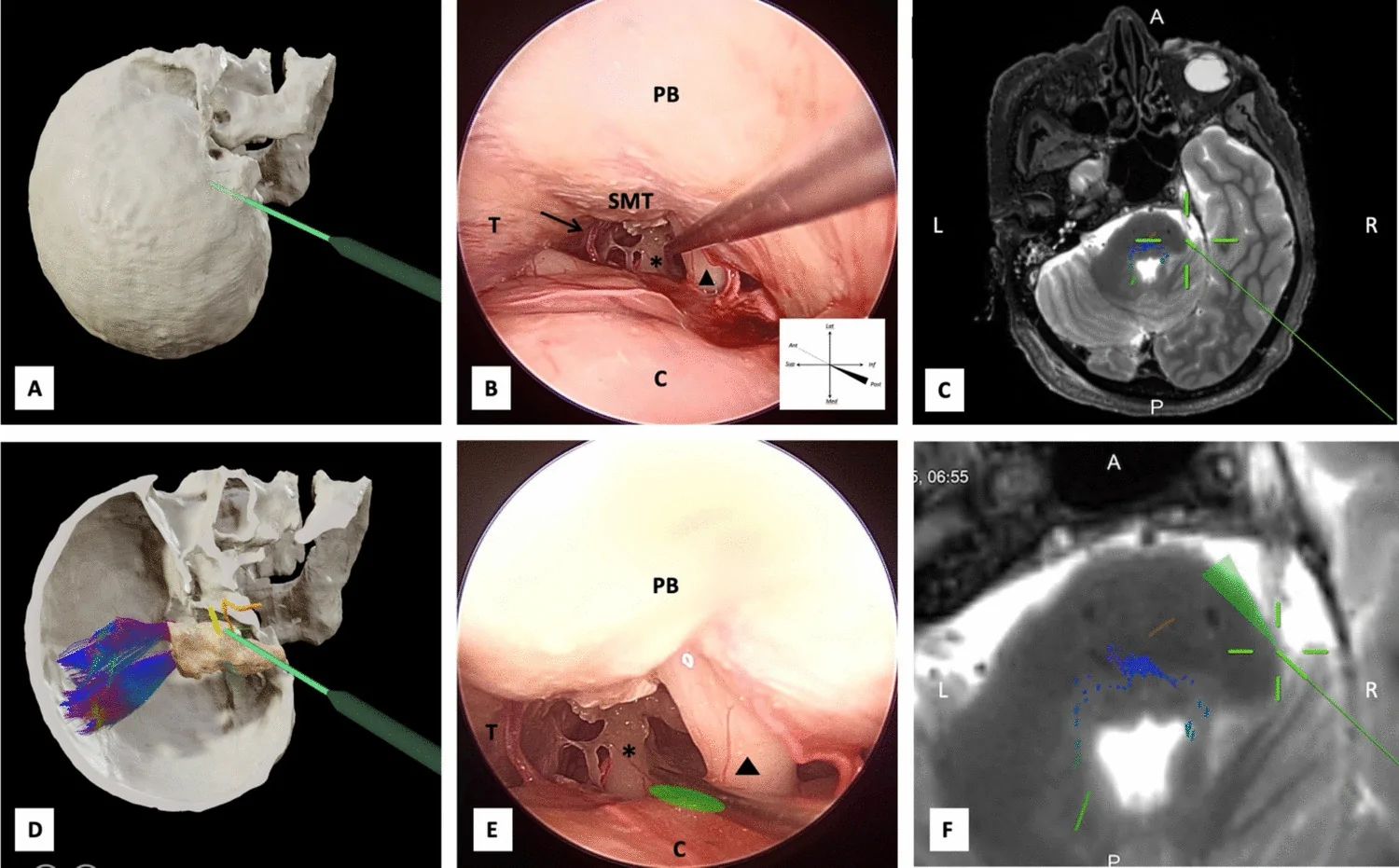
Endoscopic retrosigmoid approach. **A** 3D reconstruction of the skull prior to dissection. A neuronavigation probe (green) indicates the trajectory of the right retrosigmoid approach. **B** Arachnoid dissection extends deeply toward the facial–acoustic nerve complex (black triangle), which is the initial target, and progresses superiorly to expose the Dandy’s vein (black arrow). This allows identification of the trigeminal nerve root entry zone (REZ) in the anteromedial region (black asterisk). A prominent suprameatal tubercle may limit the viewing angle toward the trigeminal REZ. The endoscopic approach enables adequate visualization of the REZ without the need for cerebellar retraction. **C **Axial radiological image illustrating the surgical trajectory. The target point is centered on the axilla of the trigeminal nerve. **D** 3D reconstruction showing the brainstem, trigeminal nerve (yellow), facial nerve (orange), and corticospinal tract (CST) tractography. The navigation probe demonstrates the trajectory through PTZ (**E**) Close-up endoscopic view. The PTZ (green oval) is bounded by the root entry zone of the trigeminal nerve (black asterix) and the exit zone of the facial–vestibulocochlear complex (black triangle). **F** Close-up radiological view of the PTZ entry point. The trapezoidal area illustrates the direction of the intrapontine dissection. Legend: C, cerebellum; L, left; PB, petrous bone; R, right; SMT, suprameatal tubercle; T, tentorium

Intracisternal dissection toward the pons, in alignment with the endoscopic trajectory, is feasible in an infero-superior and latero-medial direction (Fig. 2).

Accessing the lateral pontine region adjacent to the abducens nerve via the **ETTA** necessitates extensive clival drilling up to the petro-clival suture. This procedure is preceded by complete skeletonization of ICA, allowing for its mobilization and lateral displacement to reach the posterolateral area in relation to the abducens nerve’s emergence.

Moreover, dural opening may increase tension on the sixth cranial nerve, and arachnoid dissection within the prepontine cistern poses a risk of injury to the perforating arteries or directly to the basilar artery.

**Fig. 3:**
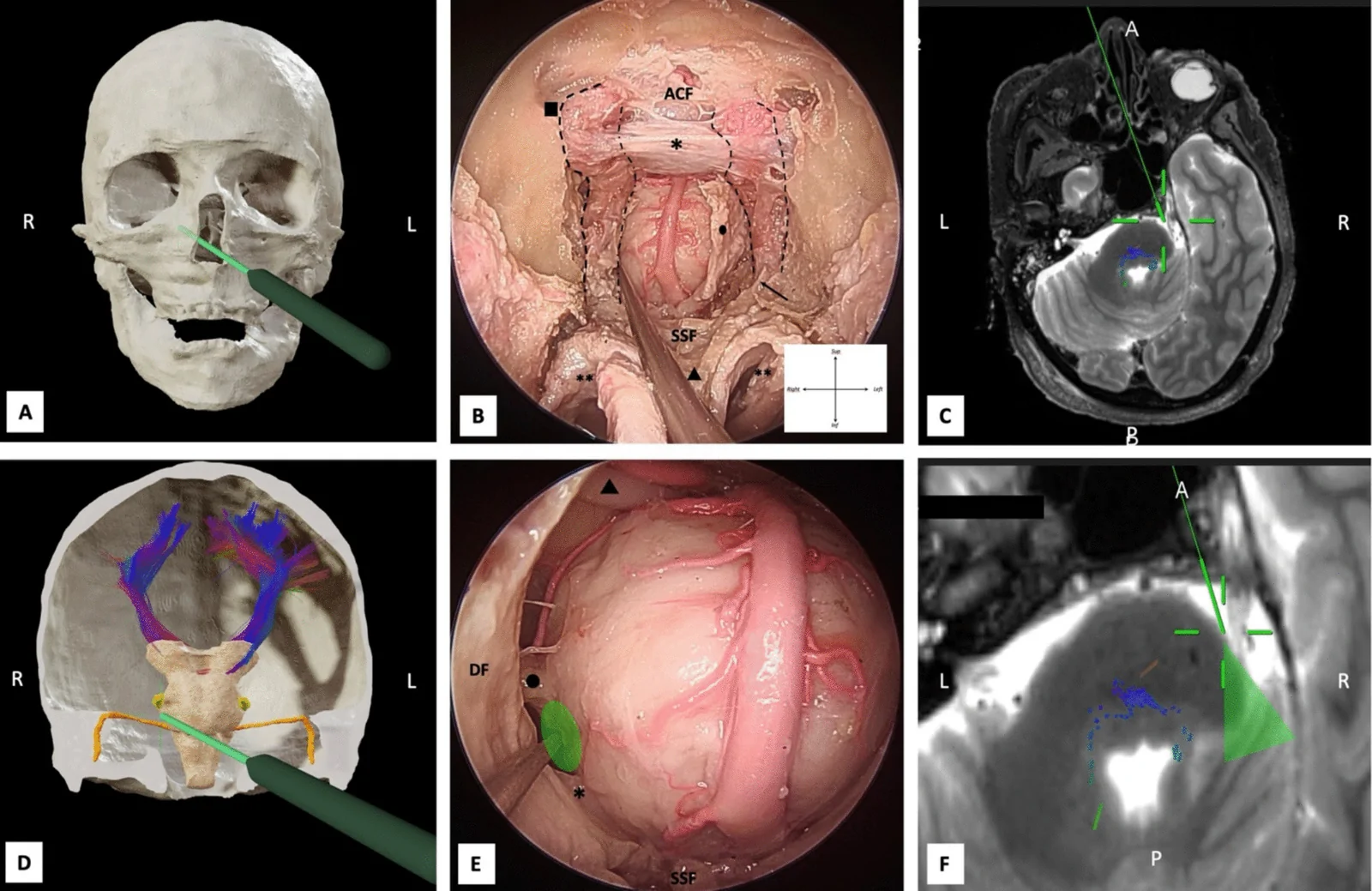
Endoscopic-transnasal-transclival approach. **A** 3D reconstruction of the skull prior to dissection. A neuronavigation probe (green) indicates the trajectory of the right transnasal-transclival approach. **B** Wide view of the sphenoid sinus. The maxillary sinuses have been removed to allow greater lateral access. The floor of the sphenoid sinus has been flattened up to the sphenoid–vomer suture to improve maneuverability (*vomer, black triangle; double asterisks, choanae*). Exposure of the sellar region aids in identifying the horizontal segments of the internal carotid arteries (ICA). The ICAs must be fully exposed to allow for lateral mobilization (highlighted with black dotted lines). The inferior limit of ICA exposure is defined by the Vidian nerve (black arrow, left side), which marks the lateral and inferior boundary of the paraclival ICA segment. The dura is opened with a Y-shaped incision: a vertical cut along the midline, and two oblique arms parallel to the floor of the sella turcica. Once the dural flaps are elevated (black dot, left side), the ventrolateral surface of the pons becomes visible. **C** Axial radiological image showing the surgical trajectory. The target is centered on the axilla of the trigeminal nerve. **D** 3D reconstruction illustrating the brainstem, trigeminal nerve (yellow), facial nerve (orange), and corticospinal tract (CST) tractography. The navigation probe demonstrates the trajectory through the PTZ. **E** Close-up endoscopic view. The PTZ (green oval) is bordered by the root entry zone of the trigeminal nerve (black dot) and the exit zone of the facial–vestibulocochlear complex, which is not directly visible using a 0-degree endoscope. The 0-degree view allows full visualization of the pons, from its inferior margin with the abducens nerve (black asterisk) to its superior limit with the oculomotor nerve (black triangle). **F** Close-up radiological view of the PTZ entry point. The trapezoidal area indicates the direction of the intrapontine dissection. Legend: ACF, anterior cranial fossa floor; DF, dural flap; L, left; R, right; SSF, sphenoid sinus floor

Despite these maneuvers, direct visualization of the PTZ via the ETTA remains constrained due to the convex profile of the brainstem, which impairs line-of-sight access to the facial-vestibulocochlear bundle exit zone. Finally, the surgical working space is restricted by the ICA and the temporal bone laterally, and by the pontine parenchyma medially (Fig. 3).

The **ETOA** enables a direct and coaxial visualization of the PTZ.

**Fig. 4:**
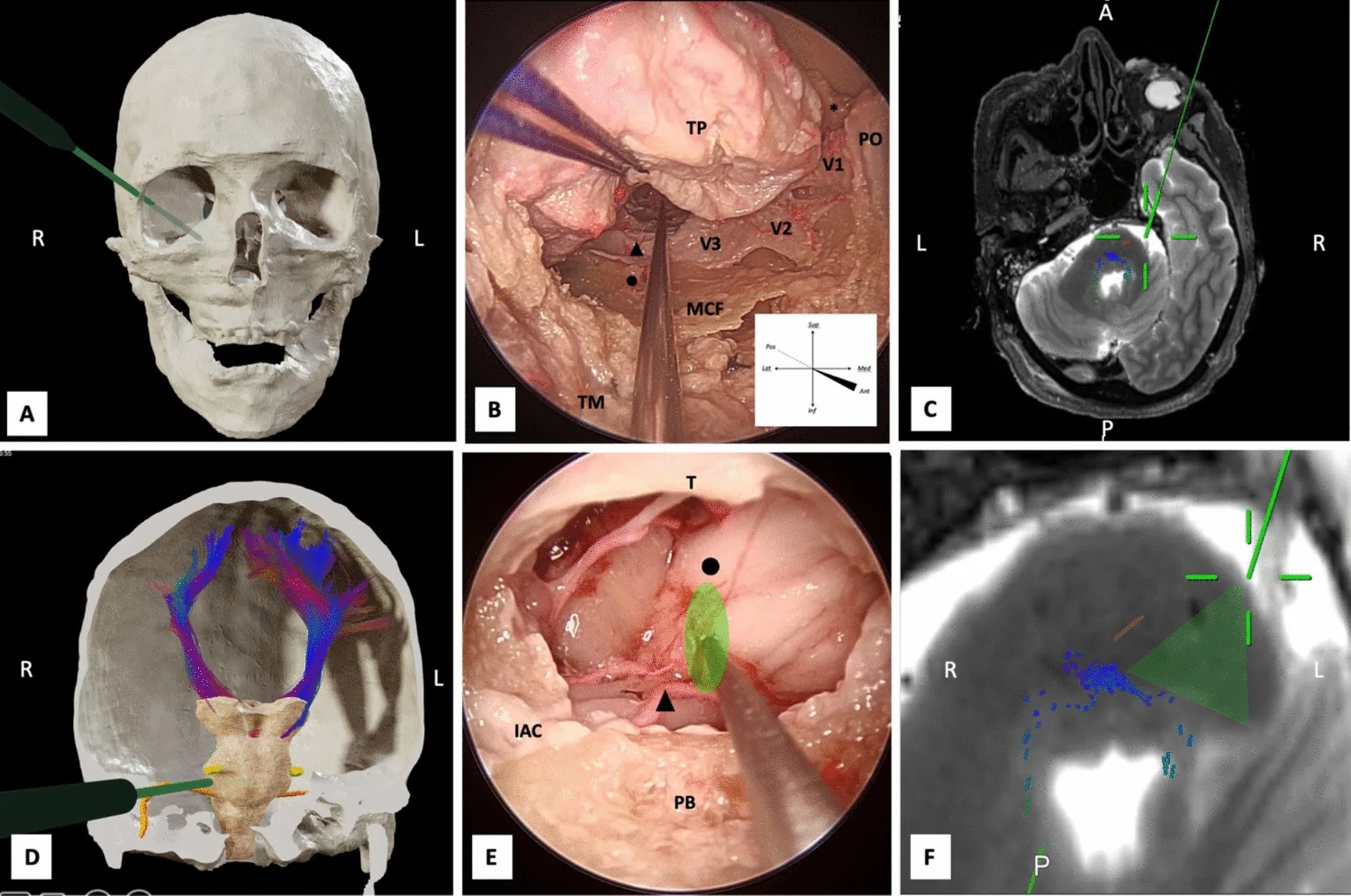
Transorbital approach. **A** 3D reconstruction of the skull prior to dissection. A neuronavigation probe (green) indicates the trajectory of the right transorbital approach. The lateral orbital rim has been removed to allow increased lateral access. The perspective is oriented inferiorly, toward the posterior cranial fossa. **B** Wide view of the middle and posterior cranial fossae. A suspension suture is applied to the dura over the temporal pole to elevate the parenchyma. The anterior portion of the greater sphenoid wing is removed to access the middle cranial fossa. The lesser sphenoid wing is completely resected, and the meningo-orbital band cut to enable interdural dissection of the lateral wall of the cavernous sinus. The "sagittal crest"—the residual bony connection between the extreme lateral portion of the lesser sphenoid wing/anterior clinoid process (asterisk) and the inferior orbital fissure—is also removed. The periorbita is visible at the level of the lateral superior orbital fissure. The floor of the middle cranial fossa is drilled and flattened to enhance maneuverability. Superior mobilization of the temporal pole is achieved by cutting the middle meningeal artery (black dot), allowing for deeper dissection to expose the petrous apex (already drilled), extending laterally to the arcuate eminence and medially to the trigeminal impression, following mobilization of the Gasserian ganglion–V3 complex. The dural fold marked with a black triangle overlies the greater superficial petrosal nerve (GSPN), which corresponds to the intrapetrous segment of the internal carotid artery (ICA), and delineates the lateral margin of Kawase’s triangle. Drilling through Kawase’s triangle grants access to the posterior cranial fossa. **C** Axial radiological image illustrating the surgical trajectory. The target point is centered on the axilla of the trigeminal nerve. **D** 3D reconstruction showing the brainstem, trigeminal nerve (yellow), facial nerve (orange), and corticospinal tract (CST) tractography. The navigation probe demonstrates the trajectory through PTZ **E:** Close-up endoscopic view. The PTZ (green oval) is bounded by the root entry zone of the trigeminal nerve (black dot) and the exit zone of the facial–vestibulocochlear complex (black triangle). **F** Close-up radiological view of the PTZ entry point. The trapezoidal area illustrates the direction of the intrapontine dissection. Legend: IAC, internal acoustic canal; L, left; MCF, middle cranial fossa’s floor; PB, petrous bone; PO, periorbita; R, right; T, tentorium; TM, temporalis muscle; TP, temporal pole; V1, first branch of the trigeminal nerve; V2, second branch of the trigeminal nerve; V3, third branch of the trigeminal nerve

Accessing the posterior cranial fossa via this route requires an anterior petrosectomy performed by drilling of Kawase’s triangle. Exposure of the petrous apex does not necessitate a complete peeling of the lateral wall of the cavernous sinus. Dissection of the dura mater lining the middle cranial fossa involves the transection of the middle meningeal artery and increases the risk of injury to the greater superficial petrosal nerve (GSPN). Although drilling and flattening of the floor of the middle cranial fossa reduce the extent of temporal lobe retraction, placing a suspension suture on the temporal pole’s dura, along with medialization of the Gasserian ganglion-mandibular branch complex provide enhanced exposure of the petrous apex. The inferolateral limit of petrous apex drilling is defined by the anterosuperior wall of the internal auditory canal. The extent of surgical maneuverability and the breadth of dissection depend on the volume of the petrous apex resected. Visualization of the PTZ can be achieved without incision of the free edge of the tentorium, thereby minimizing the risk of trochlear nerve injury (Fig. 4).

### Quantitative radiological analysis

The retrosigmoid route constitutes the shortest surgical corridor to access the PTZ, with an average working distance of approximately 67.9 ± 4.7 mm (Fig. 1). The trajectory of dissection is oriented anteriorly toward the ventral surface of the pons, thereby targeting the intrapontine segment of the corticospinal tract. Laterally, surgical maneuverability may be constrained by the presence of the suprameatal tubercle or, more generally, by the petrous portion of the temporal bone (Fig. 2). The exit point of the facial-vestibulocochlear nerve complex marks the inferolateral boundary of the surgical field, while the trigeminal REZ defines the superomedial limit.

The endonasal transclival approach represents the longest anatomical route to the PTZ, with an average operative distance of approximately 115.67 ± 13.9 mm (Fig. 1). The direction of dissection proceeds posteriorly toward the lateral pons, positioning the surgical corridor lateral to both the sixth cranial nerve and the intrapontine segment of the corticospinal tract (Fig. 3). The trigeminal nerve constitutes the superomedial boundary of the dissection trajectory, whereas the facial-vestibulocochlear complex delineates the posterolateral limit.

The endoscopic transorbital approach has an average surgical distance of approximately 90.2 ± 5.3 mm (Fig. 1). The dissection trajectory proceeds posteriorly toward the mid-pons and the fourth ventricle, positioning the intrapontine portion of the corticospinal tract medially to the operative corridor (Fig. 4). The trigeminal REZ delineates the superomedial boundary, whereas the emergence of the facial-vestibulocochlear complex defines the inferolateral limit of dissection.

One-way ANOVA revealed a statistically significant difference in the surgical corridor lengths among the three approaches (F(2, 27) = 27.41, *p* < 0.000001). Post hoc pairwise comparisons using Bonferroni correction indicated that all approaches significantly differed from each other (*p* < 0.05). Specifically, the RS approach provided the shortest mean distance, followed by the ETOA, while the ETTA demonstrated the longest surgical path to the ventral surface of the pons (Fig. 1).

The surgical angle of view to the PTZ varied significantly across the three analyzed approaches. Mean angles were 23.45° ± 3.92° for the retrosigmoid (RS) approach, 64.26° ± 4.41° for the endoscopic transorbital approach (ETOA), and 29.36° ± 3.80° for the endoscopic transtentorial approach (ETTA). A one-way ANOVA confirmed significant differences between groups (F(2,27) = 296.01, *p* < 0.00001). Post hoc pairwise comparisons with Bonferroni correction demonstrated that ETOA provided a significantly more perpendicular view to the target compared to both RS (*p* < 0.000001) and ETTA (*p* < 0.000001). Additionally, ETTA differed significantly from RS (*p* = 0.0091) (Fig. 1).

These findings indicate that the ETOA corridor enables a more direct and orthogonal trajectory toward the peritrigeminal area, potentially enhancing visualization and surgical access.

### Tridimensional analysis of the approaches

**Fig. 5:**
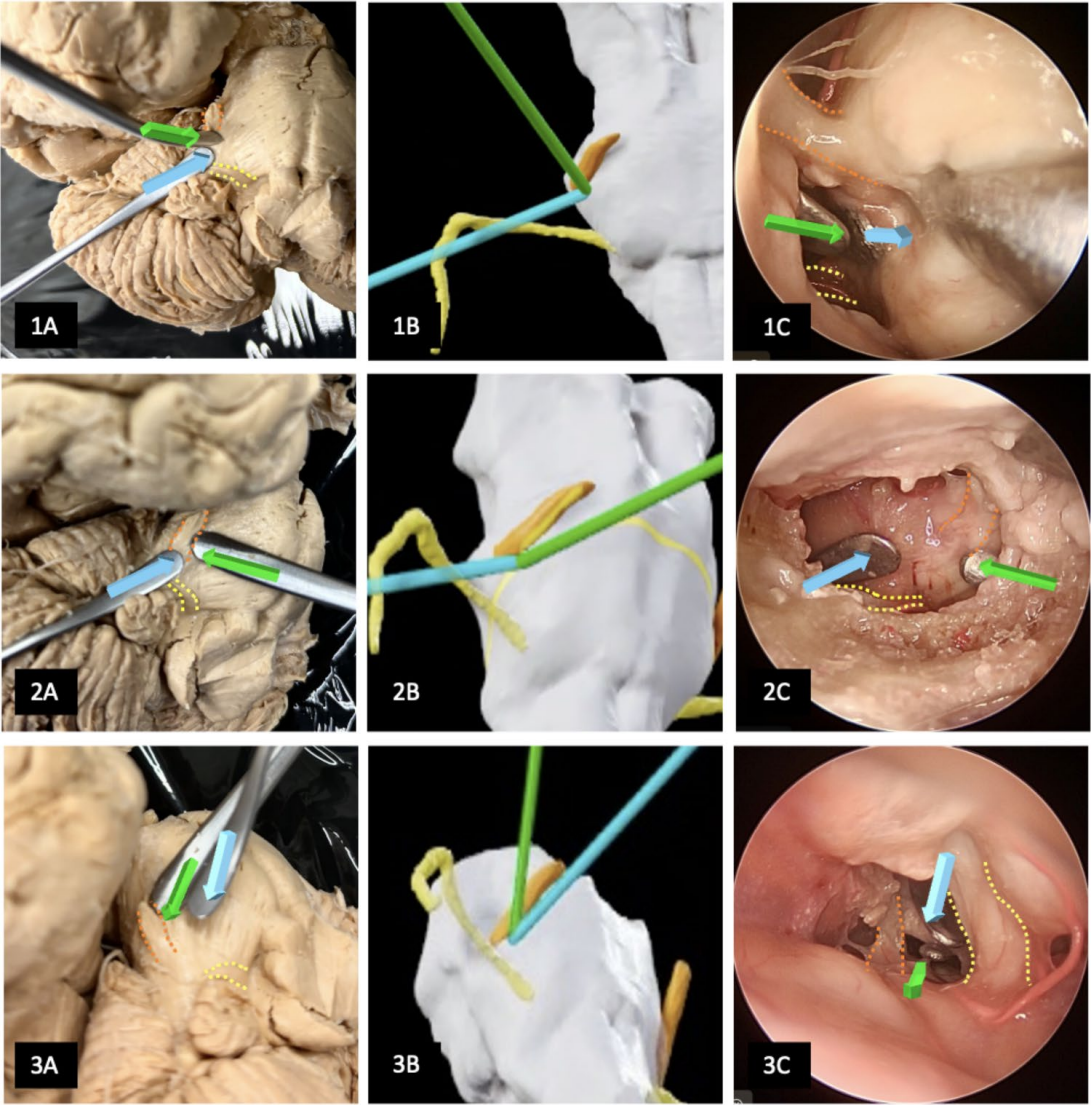
Comparative image of the three surgical approaches. The peritrigeminal zone (PTZ) is bounded laterally by the root entry zone of the trigeminal nerve (highlighted with an orange dotted line) and the root exit zone of the facial nerve (highlighted with a yellow dotted line), and medially by the corticospinal tract. The upper image series (1A, 1B, 1 C) shows the direction of the RS approach to the PTZ (blue arrow in 1 A and 1 C; blue trajectory in 1B) and the ETOA approach to the PTZ (green arrow in 1 A and 1 C; green trajectory in 1B), as viewed from the ETTA approach perspective. The middle image series (2A, 2B, 2 C) shows the direction of the RS approach to the PTZ (blue arrow in 2 A and 2 C; blue trajectory in 2B) and the ETTA approach to the PTZ (green arrow in 2 A and 2 C; green trajectory in 2B), as viewed from the ETOA approach perspective.. The lower image series (3A, 3B, 3 C) shows the direction of the ETTA approach to the PTZ (blue arrow in 3 A and 3 C; blue trajectory in 3B) and the ETOA approach to the PTZ (green arrow in 3 A and 3 C; green trajectory in 3B), as viewed from the RS approach perspective

The comparison of the surgical visualization of the PTZ through the different approaches discussed in this study provides a comparative overview of the dissection trajectories toward to PTZ (Fig. 5). As described in the previous sections, the endoscopic transnasal-transclival approach does not allow for direct visualization of the PTZ. Therefore, to depict the dissection trajectories from the other two approaches, medial displacement of the brainstem parenchyma was necessary.

The endoscopic transorbital endoscopic view highlights how the direction of dissection via both the retrosigmoid (RS) and endoscopic transnasal-transclival (ETTA) approaches runs tangentially to the pontine parenchyma. In contrast, the retrosigmoid perspective demonstrates that the trajectory of dissection through the transorbital route is perpendicular to the brainstem within the PTZ.

## Discussion

The complex neural network and the high density of nuclei within the brainstem have long driven the neurosurgical community to undertake detailed anatomical investigations, aiming to identify access routes through which surgical entry to the brainstem can be achieved with the lowest possible neurological risk. Following the pioneering efforts of early brainstem surgeons [4, 5, 30, 34] who demonstrated the relative safety of brainstem surgery, advancements in the understanding of surgical anatomy, along with technological innovations such as intraoperative neuromonitoring and high-field MRI, have enabled more refined analyses of the brainstem and the delineation of anterior, lateral, and posterior surgical corridors [6, 38].

Among these access routes, the peritrigeminal region constitutes a broad anterolateral window to the pons. This area is defined as a triangular zone, medially bordered by the pyramidal tract, laterally by a line connecting the root exit of the trigeminal nerve superiorly to the facial nerve inferiorly, and inferiorly by the pontomedullary sulcus [38, 43].

Effective surgical planning must identify the safest surface entry point, but more critically, the chosen surgical trajectory should allow maximal maneuverability to optimize surgical exposure and facilitate complete lesion resection.

The CPA route has historically been one of the most utilized corridors to access the pons [19, 40]. Consequently, the RS approach has become a cornerstone technique for brainstem surgery. However, to access more ventral or contralateral regions of the pons, extended or modified variants of the RS approach are often required—such as the extended RS approach [37], the far-lateral approach [2, 46] or the presigmoid route [22]—although some authors suggest that the standard RS approach may offer sufficient exposure of the peritrigeminal zone [19].

These posterior approaches generally follow a relatively physiological path through the CPA and provide operative access to the anteroventral pons. Nevertheless, their reach may be insufficient for controlling lesions that extend medially, toward the ventral pontine surface or posterior to the theoretical lateral boundary of the PTZ—i.e., beyond the facial and trigeminal nerves.

Anterolateral access to the pons can be achieved via transcranial approaches such as the pterional, fronto-orbito-zygomatic, or subtemporal routes, with or without anterior temporal apex drilling. However, these may not follow physiological trajectories and often necessitate substantial bony [2, 3, 6, 18, 26, 32, 39, 40].

Numerous complex approaches have now been well-characterized, and their selection is largely informed by the concept of the “angle of attack” [29, 41] and, in some cases, the "two-point method" [1, 24], which aids in identifying an optimal intrapontine resection corridor by minimizing blind spots and reducing the likelihood of incomplete resection and recurrence [17].

The development of endoscopic techniques in recent decades has further stimulated interest in minimally invasive skull base approaches that aim to reduce parenchymal retraction, bone removal, and to provide closer and more direct visualization of the lesion.

The endoscopic approach, characterized by its tubular access path, has inspired the evolution of the “one-point method,” which builds upon the two-point concept by incorporating identification of the closest safe entry zone and radiological insights from DTI-based MRI sequences [35].

Among the most familiar endoscopic approaches, the transnasal-transsphenoidal route with clival extension is notable. Studies by Weiss et al. and D’Avella et al. demonstrate that reaching the PTZ via a transnasal route necessitates lateral clival drilling up to the temporal apex and full skeletonization of ICA and the sixth cranial nerve [9, 43]. Although anterior petrosectomy via this route is feasible, it remains highly invasive for direct intrapontine lesion removal via the PTZ [9, 20, 23].

Among the newer endoscopic corridors, the transorbital approach has emerged as a safe and effective alternative for PTZ access in skilled hands. Following confirmation of anterior petrosectomy feasibility [14, 21, 28], the transorbital route adopts a pretemporal path, offering an anterolateral cranial-to-pons trajectory. This allows coaxial dissection along the lesion's long axis, minimizing the surgical angle of attack.

This study compares access corridors specifically targeting the posterior PTZ—namely, the region between the exiting and entering rootlets of the facial and trigeminal nerves, respectively. Lesions with postero-medial to anterolateral development within this region may be only partially resectable when approached via RS or ETTA due to a reduced angle of attack.

Anatomical and radiological imaging revealed that the CPA route via the RS offers a dissection trajectory extending from the posterior PTZ anteriorly toward the intrapontine pyramidal tract. However, this approach appears to be theoretically limited for lesions extending posteriorly beyond the exit zones of the facial and trigeminal nerves, which thus represent the posterior dissection boundary due to the near absence of surgical visibility.

In contrast, the ETTA facilitates anterior PTZ access and provides an increasing distance from the pyramidal tracts. However, it lacks full visualization of the facial-acoustic bundle. Additionally, the surgical trajectory is long and tangential relative to the brainstem, which may constrain maneuverability toward more medial pontine areas.

The ETOA, on the other hand, affords a nearly perpendicular trajectory toward the pons, enabling direct visualization of the fifth and seventh cranial nerves. Its anterior dissection limit is defined by the Gasserian ganglion–mandibular nerve complex, preceding the pyramidal tracts. As with the retrosigmoid route, the ETOA provides comprehensive exposure of the trigeminal and facial nerve root exits, maintaining clear visualization of the PTZ’s posterolateral boundary.

While the CPA route from a RS approach requires minimal bone removal and offers a narrower corridor, it often necessitates cisternal decompression or suprameatal tubercle drilling to improve maneuverability.

ETTA, despite internal carotid skeletonization for lateral pons exposure, involves a long working distance, lateralization challenges, and restricted instrument mobility.

Conversely, the ETOA yields a direct view and satisfactory maneuverability within the PTZ, with a shorter corridor than ETTA; however, partial cavernous sinus peeling and Kawase’s triangle drilling increase surgical comorbidities.

In the context of clinical application, the choice of surgical corridor to the PTZ should be guided not only by anatomical feasibility but also by the specific pathophysiological characteristics of the lesion. The RS approach remains particularly suitable for lesions located within or extending toward the cerebellopontine angle, such as intrinsic pontine cavernous malformations or exophytic gliomas, where a posterior route allows for adequate exposure with limited bony removal. Conversely, the ETTA approach is best indicated for midline ventral or ventrolateral pathologies—including chordomas, chondrosarcomas, and other clival-based neoplasms—that require anterior access without significant cerebral and cerebellar retraction. The ETOA route, although technically demanding, offers distinct advantages for lesions of the anterolateral pons and petroclival junction, including selected intrapontine cavernomas and neoplastic processes with a lateral or oblique growth pattern. Its near-perpendicular trajectory facilitates dissection along the major axis of such lesions, thereby enhancing the potential for gross total resection. Overall, the integration of lesion morphology, growth trajectory, and neurovascular relationships remains essential in determining the most appropriate approach, emphasizing that no single corridor is universally optimal but rather context-dependent.

In conclusion, although all approaches assessed in this study offer substantial exposure of the peritrigeminal region, each presents theoretical limitations and associated morbidities. A comprehensive, circumferential understanding of the peritrigeminal anatomy and corresponding radiological representations provides essential guidance to skull base neurosurgeons, facilitating more informed and deliberate access to the anterolateral brainstem.

## Study limitations

Several methodological limitations must be considered when interpreting the translational relevance of these findings.

The lack of physiological variables such as blood flow, CSF, tissue consistency, and retraction tolerance limits the ability to replicate live surgical conditions.

This study is based on the evaluation of the PTZ through different bony corridors. However, the lack of basal cistern turgor resulting from the absence of CSF in cadaveric specimens, unlike in live surgical conditions, inherently limits the ability to expand the viewing and working angles toward the surgical target.

Quantitative evaluation of approach length and surgical viewing angle was performed on the specimens prior to dissection. Future investigations will focus on the quantitative assessment of the exposed area following the execution of the approaches, to provide a more accurate estimation and comparison of the effective surgical exposure.

Moreover, the limited sample size may fail to capture the anatomical variations among individuals, so it restricts the applicability of the anatomical observations.

Finally, DTI-based tractography in post-mortem specimens is subject to reduced accuracy due to altered diffusivity profiles, which compromises the precision of probabilistic mapping of intrapontine white matter pathways.

## Conclusions

The comparative analysis of endoscopic approaches to the PTZ has enabled a comprehensive anatomical understanding of this pontine area. Both the RS and ETTA provide tangential access to the PTZ, with dissection trajectories oriented posterolaterally for ETTA and anteromedially for RS. In contrast, the ETOA offers a more perpendicular trajectory, facilitating an intrapontine dissection path that is more obtuse relative to the surface entry point. Each of these approaches carries inherent procedural limitations that must be carefully evaluated in clinical decision-making. Increasingly refined anatomical knowledge and advanced surgical expertise in the era of endoscopic-assisted surgery are essential to minimizing morbidity in procedures targeting such a deep, central, and functionally critical neuroanatomical region.

## Data Availability

No datasets were generated or analysed during the current study.
